# Cardiovascular Involvement in Pediatric Laminopathies. Report of Six Patients and Literature Revision

**DOI:** 10.3389/fped.2020.00374

**Published:** 2020-07-24

**Authors:** Anwar Baban, Marianna Cicenia, Monia Magliozzi, Maria Gnazzo, Nicoletta Cantarutti, Massimo Stefano Silvetti, Rachele Adorisio, Bruno Dallapiccola, Enrico Bertini, Antonio Novelli, Fabrizio Drago

**Affiliations:** ^1^The European Reference Network for Rare, Low Prevalence and Complex Diseases of the Heart-ERN GUARD-Heart, Pediatric Cardiology and Arrhythmia/Syncope Units, Bambino Gesù Children Hospital and Research Institute, Rome, Italy; ^2^Laboratory of Medical Genetics, Bambino Gesù Children Hospital and Research Institute, Rome, Italy; ^3^The European Reference Network for Neuromuscular Disorders (ERN NMD), Unit of Neuromuscular and Neurodegenerative Disorders, Bambino Gesù Children Hospital and Research Institute, Rome, Italy

**Keywords:** *LMNA* variants, laminopathy, arrhythmias, dilated cardiomyopathy, congenital heart defects, aortic coarctation

## Abstract

Lamin A/C (*LMNA*) encodes for two nuclear intermediate filament proteins. Mutations in *LMNA* cause a highly heterogeneous group of diseases predominantly leading to muscular or cardiac disease, lipodystrophy syndromes, peripheral neuropathy, and accelerated aging disorders. Cardiac involvement includes progressive arrhythmias (brady/tachyarrhythmias, sudden cardiac death). Furthermore, cardiomyocyte damage often progresses into dilated cardiomyopathy (DCM), rarely described in the pediatric age group. Neuromuscular manifestations are even rarer in children. We report on six pediatric patients with *LMNA* mutations: patient 1 was operated on for aortic coarctation, non-compact left ventricle, atrial fibrillation (AF) preceding the diagnosis of DCM; patient 2 was operated on for ventricular septal defect (VSD), developed after years malignant arrhythmias preceding the progression to DCM (left ventricular non-compaction with LV dysfunction); patient 3 had ectopic atrial tachycardia as first manifestation of a DCM; patients 4 and 5 had no major arrhythmic events but only dilated ascending aorta, mildly dilated LV with mild hypertrabeculation of the lateral wall and a normally functioning but dilated left ventricle, respectively; patient 6 showed aortic coarctation, supraventricular tachycardia. Paroxysmal AF occurred in patients 1, 2, and 3 (50% of cases). Our series highlight the coexistence of congenital heart defects (CHDs) and aortic involvement with laminopathies in four of our patients: consisting of aortic coarctation (two patients), aortic root dilatation (one patient), and VSD (one patient). Aortic changes in laminopathies have been reported only once in an adult patient. This is the first report in the pediatric setting, and no associations with CHD have been previously described.

## Introduction

*LMNA* (OMIM ^*^150330) is located on chromosome 1q21.22 (1) and includes 12 exons. Five A-type lamins (A, AD10, AD150, C, and C2) are encoded and are produced by alternative splicing. Lamins A and C are the two major isoforms, and the latter has the highest expressed transcript (2, 3).

*Nuclear lamina* is the term applied to the ubiquitous nuclear intermediate filament proteins. Nuclear stability is offered through the binding process to a major number of nuclear protein complexes. Moreover, they help connect the nucleus to the cytoskeleton and contribute to genome stability, differentiation, modulate chromatin organization, gene regulation and expression, and tissue-specific functions (4, 5).

Lamins A and C are widely expressed in skeletal and cardiac muscle. However, it is well-expressed as well in fat, blood vessels, skin, and nerve tissue (6).

LMNA variants cause a series of rare and diverse diseases called laminopathies (7). The numerous roles of the lamina in which it is involved explain the wide range of diseases for which it can be responsible.

Worman and Bonne (8) suggest four major classes of laminopathies according to major presenting signs and symptoms: diseases of striated and cardiac muscle, lipodystrophy syndromes, peripheral neuropathy, and premature aging. Currently, there are at least 12 clinically distinct disorders that show disease-specific variants in LMNA (8).

Dilated cardiomyopathy (DCM) is the most common form of cardiomyopathy (CMP) and is associated with cardiac dilatation and impaired systolic function. It is one of the major causes of sudden cardiac death (SCD). Variants in more than 60 genes are associated with idiopathic DCM. *LMNA* variants range from 5 to 10% of familial DCM and 2 to 5% of sporadic DCM (~7% of all idiopathic DCM cases) (9–12). *LMNA*-related CMP are frequently associated with both tachyarrhythmias and bradyarryhthmias. They are commonly associated to supraventricular arrhythmias and conduction delay and less commonly to ventricular tachyarrhythmias and eventually leading to SCD.

Cardiac involvement in adult-onset laminopathies including Emery–Dreifuss and limb girdle muscular dystrophy is well-described, but given the relatively recent description and rarity of laminopathies, there are few data on age at onset, cardiac progression, risk of arrhythmias, and SCD in the pediatric population (13, 14).

Here we describe our experience in the field of laminopathies in pediatric population showing phenotypes ranging from arrhythmias and DCMs to congenital heart defects (CHDs).

## Materials and Methods

We reviewed medical records of patients with *LMNA* variants seen in our tertiary care center. This is a single-center, observational, both retrospective and prospective analyses. All data, including the cardiac diagnosis and surgical reports, were extracted from our cardiac database in the period from 2015 to 2019, case notes, reports of echocardiography and catheterization, and operative notes.

### DNA Analysis

Genomic DNA was extracted from blood leukocytes using standard procedures. Mutational analysis of our cardiogenetic panel genes was performed by a custom-panel design prepared using Design Studio software high-throughput Nimble Gene SeqCap EZ Custom Enrichment Kit (Roche Life Science, Mannheim, Germany) through Illumina (Next Seq 550) (San Diego, CA, USA) platform based on GRCh37 genome (H sapiens, hg19). The target parameters were the coding exons including a region extension of 25 bases from the 3′ end and 25 bases from the 5′ end (based on RefSeq database). We obtained a targeted next-generation sequencing (NGS) assay that has a mean 100× coverage for >97% bases, a specificity of 100%, and sensitivity of 100%, with a quality score of ≥30. Sequencing was performed using an Illumina sequencer (MiSeq and NextSeq550), and a bioinformatics program (Variant Studio; Illumina) was concurrently used to assess the data concerning the implementation of the NGS technology. Each variant identified has been evaluated for call quality score and coverage and visualized by Integrative Genome Viewer. All variants identified were validated by Sanger sequencing using standard protocols. The core gene panel that we mainly examine in our clinic included the analysis of the following genes: *ABCC9* (*NM_020297.2*), *ACTA2* (*NM_001613*), *ACTC1* (*NM_005159.4*), *ACTN2* (*NM_001103*), *ANKRD1* (*NM_014391*), *BAG3* (*NM_004281*), *CAV3* (*NM_001234*), *CITED2* (*NM_006079.4*), *COL3A1* (*NM_000090*), *CTNNA3* (*NM_013266*), *CRELD1* (*NM_001031717.3*), *DES* (*NM_001927.3*), *DSC2* (*NM_024422*), *DSG2* (*NM_001943*), *DSP* (*NM_004415*), *ELN* (*NM_000501*), *FBN1* (*NM_000138*), *FBN2* (*NM_001999*), *FLNC* (*NM_001458*), *GATA4* (*NM_002052.4*), *GATA6* (*NM_005257.5*), *GJA1* (*NM_000165.4*), *GJA5* (*NM_005266.6*), *GLA* (*NM_000169*), *ISL1* (*NM_002202.2*), *JAG1* (*NM_000214.2*), *JUP* (*NM_002230*), *LAMP2* (*NM_013995*), *LDB3* (*NM_007078*), *LMNA* (*NM_170707*), *MED13L* (*NM_015335.4*), *MYBPC3* (*NM_000256*), *MYPN* (*NM_032578*), *MYH6* (*NM_002471*), *MYH7* (*NM_000257*), *MYH11* (*NM_002474*), *MYL2* (*NM_000432*), *MYL3* (*NM_000258*), *MYLK* (*NM_053025*), *NEXN* (*NM_144573*), *NKX2.5* (*NM_004387.3*), *NKX2.6* (*NM_001136271.2*), *PKP2* (*NM_004572*), *PLN* (*NM_002667*), *PRKAG2* (*NM_0162039*), *RBM20* (*NM_001134363*), *SLC2A10* (*NM_030777*), *SMAD3* (*NM_005902*), *SMAD6* (*NM_005585*), *TAZ* (*NM_000116*), *TBX20* (*NM_001077653.2*), *TCAP* (*NM_003673*), *TGFB2* (*NM_003238*), *TGFB3* (*NM_003239.2*), *TGFBR1* (*NM_004612*), *TGFBR2* (*NM_003242*), *TMEM43* (*NM_024334*), *TNNC1* (*NM_003280*), *TNNI3* (*NM_000363*), *TNNT2* (*NM_000364*), *TPM1* (*NM_001018005*), *TTR* (*NM_000371*), *SCN5A* (*NM_198056*), *VCL* (*NM_014000*), *ZFPM2* (*NM_012082.3*). *TTN* truncating mutations (NM_001267550.1).

### Literature Review

We performed a review of previous studies describing the coexistence of *LMNA* variant and cardiac involvement in the pediatric population. We searched PubMed for published studies with no restriction on date of publication and no restriction on language, using the following search terms: “*LMNA* MUTATION” AND “CHILDREN” AND “CARDIAC INVOLVEMENT” or “*LMNA* MUTATION” AND “PEDIATRIC POPULATION” AND “CARDIAC DISEASE.” We included all types of studies, provided that the study population encompassed at least one patient with laminopathy diagnosed at age younger than 18 years and concomitant cardiac disease. Full articles were carefully read and reconsidered according to the aforementioned criteria. Two investigators performed the search independently. References of selected articles were crosschecked with the same inclusion condition. Duplicates were removed.

### Editorial Policies and Ethical Considerations

The study protocol conforms to the ethical guidelines of the 1975 Declaration of Helsinki, as reflected in *a priori* approval by the institution's Human Research Committee. We confirm that informed consent has been obtained from the parents of our probands or by the proband himself/herself.

### Cases Presentation

**Figure 1:**
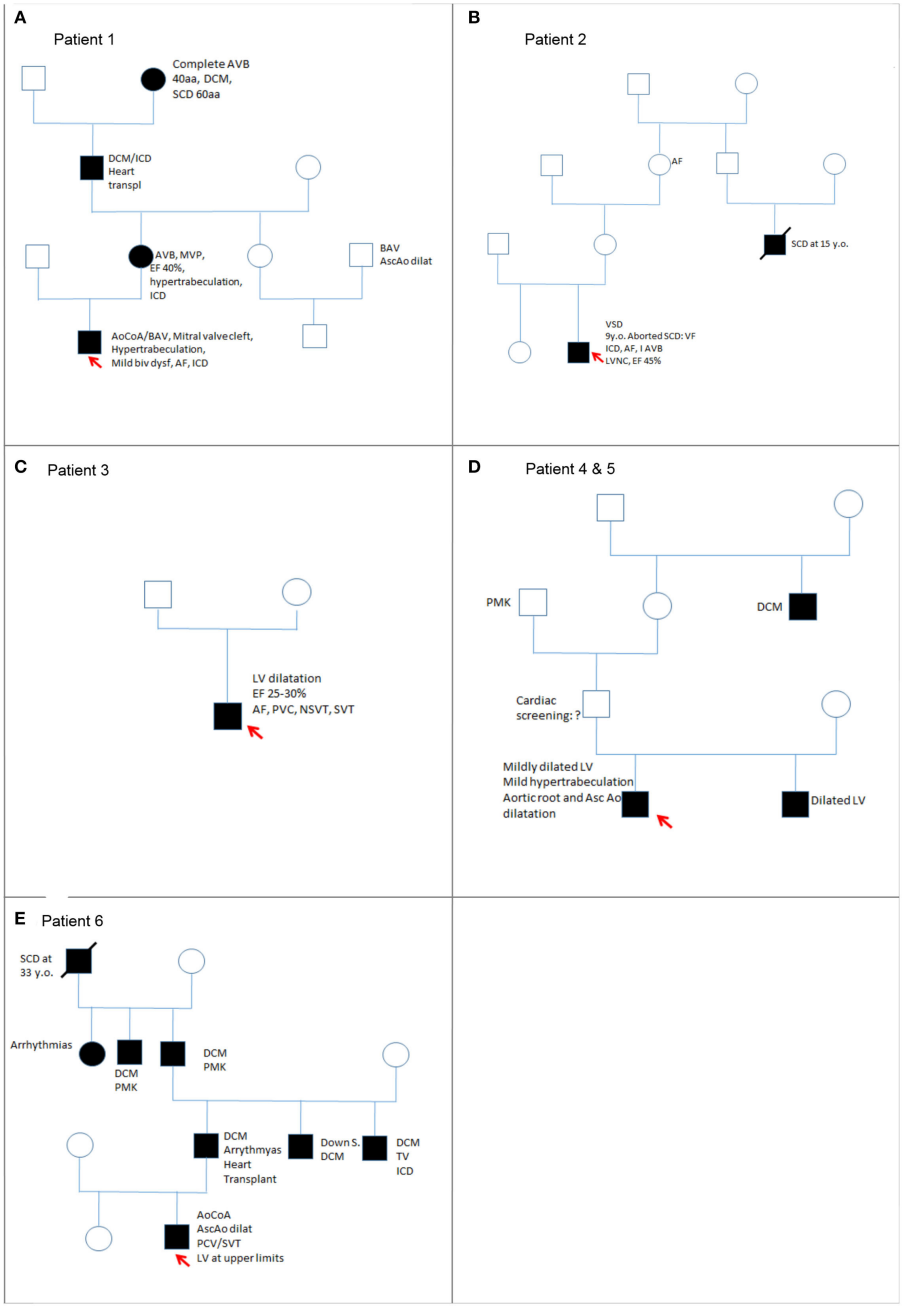
Family pedigrees (from **A–E**) of the cohort of children with *LMNA* variants.

Table 1 includes a summary of main clinical features of this cohort, whereas Figure 1 includes the family pedigrees of the cohort.

**Table 1:** Main characteristics of our pediatric patients affected by laminopathies presenting with cardiac disease.

	**Patient 1**	**Patient 2**	**Patient 3**	**Patient 4**	**Patient 5**	**Patient 6**
LMNA variant	c.673C>T (p.Arg225Ter)	c.1201C>T (p.Arg401Cys)	c.746G>A (p.Arg249Gln)	c.598A>G (p.Met200Val) (brother of patient 5)	c.598A>G (p.Met200Val) (brother of patient 4)	c.214delC (Arg72Alafs*24)
Inheritance	Maternally inherited (affected), mother, grandfather and great grandmother: CMP + arrhythmia	Maternally inherited (maternal line SCD)	*De novo*	Paternally inherited (Vous) paternal uncle: CMP (onset <1 year old), paternal grandfather: CMP	Paternally inherited (Vous) paternal uncle: CMP (onset <1 year old), paternal grandfather: CMP	Paternally inherited (affected). Father, >1 paternal uncle, grandfather: CMP.
First cardiac event	Aortic coarctation Bicuspid aortic valve Mitral valve cleft	Repaired VSD, post-operative RBBB	Ectopic atrial tachycardia myocarditis	Mildly dilated LV; mild hypertrabeculation of the lateral wall; dilatation of the aortic root and an ascending aorta at the higher limits of the normality	Dilated LV with preserved EF	Aortic coarctation
Endpoints	*Paroxysmal* AF Mild biventricular dysfunction *S/P ICD implant (primary prevention)*	VF; S/P ICD LVNC-EF 45% I degree AVB *Paroxysmal* AF	DCM, *LBBB*	–	–	Redundant mitral valve Upper normal limits LV Ascending aorta and sinotubular junction dilatation EVB, SVT
Age at CHD diagnosis	At birth	At birth	No CHD	No CHD	No CHD	At birth
Age at first manifestation of HF/rhythm disturbances	11 years old	9 years old	13 years old	11 years old	3 years old	17 years old
Neuromuscular and general phenotypic aspects	III and V fingers camptodactyly; hip asymmetry; sloping shoulders; turricephaly; short and down-slanting palpebral fissures; prominent but low-set ears; asymmetry of mandibular bite and micrognathia	Down-slanting palpebral fissures; prominent but low-set ears; asymmetry of mandibular bite; micrognathia; long neck; sloping shoulders; pectus excavatum	Normal	Mild skeletal anomalies	Mild skeletal anomalies	Short palpebral fissures; prominent ears; jaw asymmetry; he has a scoliosis; right-sided hemihypertrophy; curved vara knees

*AF, atrial fibrillation; ICD, implantable cardiac defibrillator; HF, heart failure; LVNC, left ventricular noncompaction; EF, ejection fraction; VSD, ventricular septal defect; AVB, atrium-ventricular block; RBB, right bundle-branch block; DCM, dilated cardiomyopathy; EVB, ectopic ventricular beats; SVT, supraventricular tachycardia; Vous, variant of unknown significance*.

### Patient 1

This patient, 16 years old, came to our attention for his medical history of aortic coarctation, bicuspid aortic valve and mitral valve cleft. Family history was positive for both cardiomyopathies and CHDs. His great grandmother (mother of maternal grandfather) was affected by DCM since the age of 40 years; she received pacemaker (PMK) and died suddenly at the age of 60 years. His maternal grandfather received the diagnosis of DCM at 40 years old; he underwent implantable cardiac defibrillator (ICD) implantation and finally heart transplantation at age 60 years because of refractory heart failure. He died shortly from post-operative complications.

At the age of 44 years, the mother showed first-degree AVB at electrocardiogram (ECG), mitral valve prolapse, moderate ventricular dysfunction [ejection fraction (EF) 40%], and hypertrabeculation of the left ventricle (LV). Cardiac magnetic resonance (CMR) showed no signs of edema and thin late gadolinium enhancement (LGE) intramyocardial layer at medium–basal level of the LV. She started treatment with β-blockers and received an ICD. His cousin (son of mother's sister) had a bicuspid aortic valve and dilatation of the ascending aorta.

**Figure 2:**
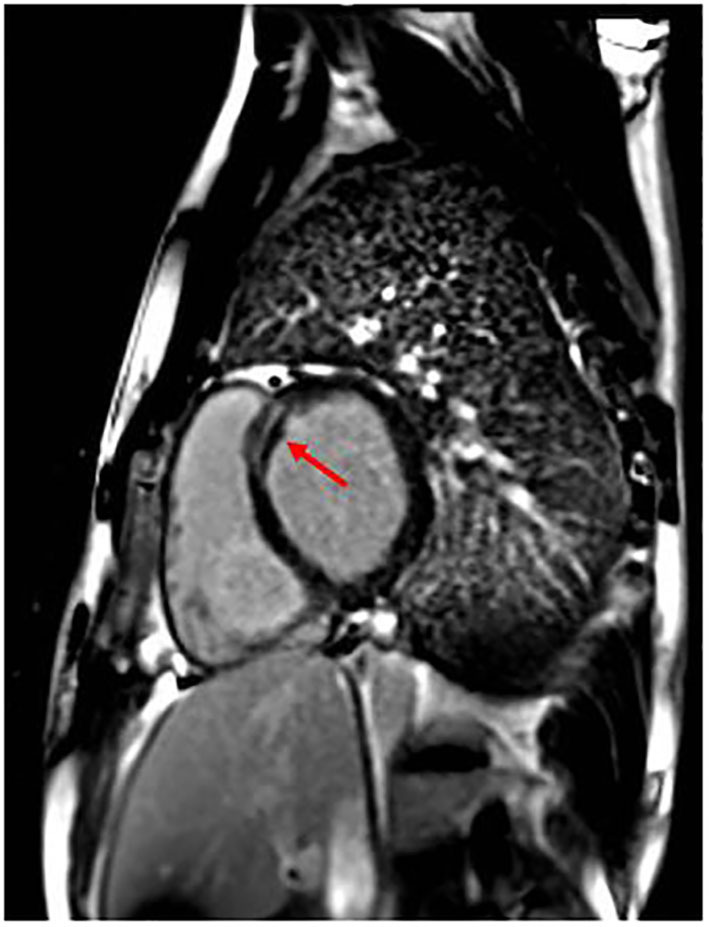
Patient 1 focal image of the cardiac magnetic resonance imaging (MRI) in short-axis view showing LGE at the intramyocardial wall at the medium–basal level of the interventricular septum. The red arrow indicates the areas with major involvement of LGE (late gadolinium enhancement).

The patient was operated on for coartectomy at 5 months old. At the age of 9 years, a recoarctation was detected that needed an aortic stent implantation. Two years later, he developed paroxysmal atrial fibrillation (AF) episodes successfully treated with DC shock (one) and intravenous flecainide (another one); other self-terminating episodes were prevented with antiarrhythmic drugs (flecainide and nadolol). Moreover, CMR showed initial signs of biventricular systolic dysfunction: globular-shaped LV, at the upper limit of normal (end-diastolic volume 198.5 mL, indexed end-diastolic volume 96.6 mL/m^2^, end-systolic volume 105 mL, indexed end-systolic volume 51.1 mL/m^2^) and mildly dilated when compared to the right ventricle (LV end-diastolic volume/right ventricle end-diastolic volume 1.1; reference value, 0.9), with LVEF 47%; mild systolic dysfunction of the right ventricle (EF 48%); and presence of LGE of intramyocardial wall at the medium–basal level of the interventricular septum (Figure 2). Because of these findings and family history, the patient received ICD implantation for primary prevention. Creatine kinase (CK) concentration was mildly elevated only once (total measurements, 3). He also suffered from epileptic attacks started at age 10 years. He was operated on for severe bilateral flat feet. The patient had normal neuromuscular development and motor skills. However, specific musculoskeletal evaluation showed camptodactyly of III and V fingers of the hands, a mild hip asymmetry, sloping shoulders, and turricephaly. At facial level, he had short and down-slanting palpebral fissures, prominent but low-set ears, asymmetry of mandibular bite, and micrognathia.

Cardiomyopathy panel analysis showed a maternally inherited heterozygotic variant c.673C>T of the *LMNA*, determining p.Arg225Ter.

### Patient 2

The medical history of this patient started during his first year of life when he was operated on for a ventricular septal defect (VSD) without complications. In his family, the mother's cousin died suddenly at age 15 years, and his maternal grandmother was affected by AF since 60 years old.

The continued regular follow-up showed appropriate anatomical repair and regular sinus rhythm with right bundle-branch block (QRS duration 125 ms). At the age of 9 years, he had cardiac arrest due to ventricular fibrillation (VF) while having a bath, and an ICD was implanted as secondary prevention. After 3 years, another VF was successfully treated by an appropriate shock. The echocardiogram showed LV non-compaction (LVNC) and mild ventricular dysfunction (EF 45%). The diagnosis of LVN was made in accordance with the echocardiographic criteria of Jenni et al. (15) and Chin et al. (16). Later, he developed first-degree AVB. At 19 years old, he had self-limited paroxysmal episodes of atrial flutter/fibrillation treated with sotalol. Creatine kinase level was normal. Facial dysmorphisms included down-slanting palpebral fissures, prominent but low-set ears, asymmetry of mandibular bite, and micrognathia; long neck, sloping shoulders, and pectus excavatum were present.

Considering his personal and familial medical history, genetic analysis was performed revealing maternally inherited heterozygotic variant in *LMNA*, c.1201C>T (p.Arg401Cys). Maternal workup was not performed.

### Patient 3

His medical history started at 13 years old when he was diagnosed with myocarditis due to parvovirus B19 identified at endomyocardial biopsy (EMB). Family history was negative.

**Figure 3:**
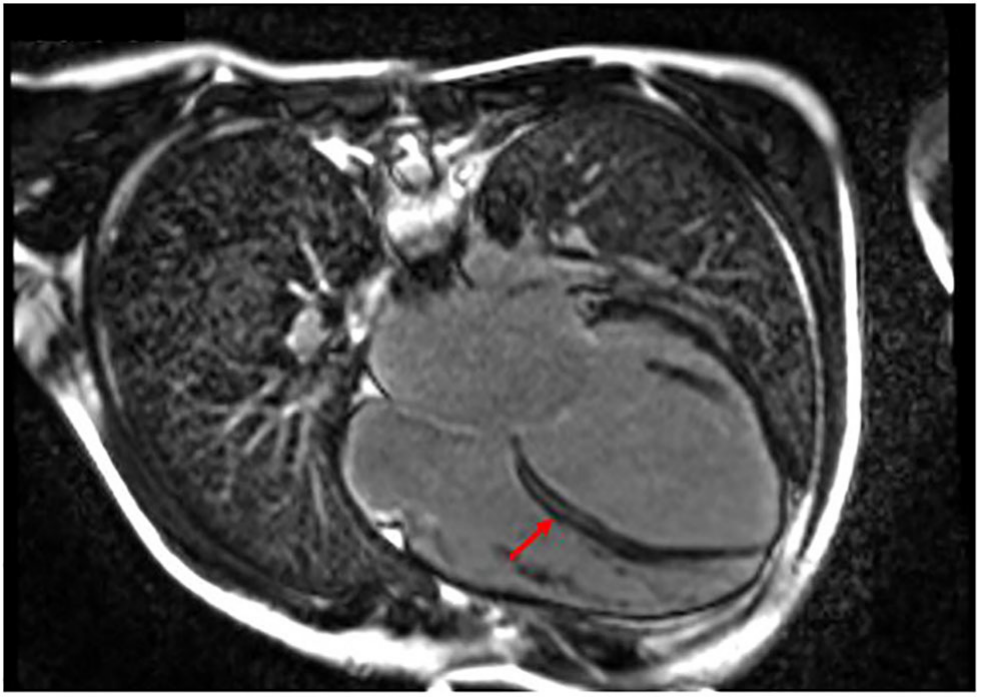
Patient 3 focal image of cardiac MRI in a 4-chamber view showing LGE with midwall fibrosis of the interventricular septum at medium–basal level of the basal inferior wall. The red arrow indicates the areas with major involvement of LGE (late gadolinium enhancement).

After a short period of partial recovery (LVEF 40–45%), he developed ectopic atrial tachycardia (EAT) and severe LV dysfunction (LVEF 25–30%), and myocarditis relapse was suspected. Cardiac magnetic resonance was performed twice and in both cases showed a layer of midwall fibrosis of the Interventricular septum (IVS) at medium–basal level and of the basal inferior wall (Figure 3); no edema was detected. The second EMB showed the presence of inflammation with lymphomonocytic infiltration, but without evidence of cytotoxic effects. Creatine kinase was constantly and remarkably increased (minimum value, 736 UI/L; maximum value, 1,273 UI/L; reference range, 39–308 UI/L). Neuromuscular development was normal. Serial ECG and Holter recordings demonstrated stable first-degree AVB, complete LBBB, polymorphic premature ventricular complex (PVC) means extrasystolic beat, non sustained ventricular tachycardia (NSVT) is a tachycardia lasting less than 30 seconds, and sustained ventricular tachycardia (SVT) is a tachycardia lasting more than 30 seconds. Symptomatic paroxysmal AF occurred and was treated with intravenous and oral amiodarone. The genetic analysis identified a *de novo*, heterozygotic variant c.746G>A of the *LMNA*, coding for the previously reported protein variant p.Arg249Gln.

### Patients 4 and 5

Patient 4 was in follow-up for normally functioning but mildly dilated LV and mild hypertrabeculation of the lateral wall since he was 11 years old. He also had dilatation of the aortic root and an ascending aorta at the upper limits of normal (aortic root *z* score +4.2 and ascending aorta *z* score 2.1 at the last follow-up). Creatine kinase was normal. He presented with poor growth (at lower normal limits for age) and dolichostenomelic habitus, thin and soft skin, pectus excavatum, mild hyperlaxity, spindle-shaped fingers, sunken eyes with down-slanting palpebral fissures, thin nasal bridge with pinched tip and hypoplasia of nasal ali, narrow palate, and overcrowding of teeth.

His brother (patient 5), presented at age 3 years, had a normally functioning but dilated LV (end-diastolic diameter LV *z* score + 3) with a preserved EF and only mild skeletal abnormalities microsomy with dolichostenomelic habitus, thin skin, pectus excavatum, mild hyperlaxity, spindle-shaped fingers, sunken eyes with down-slanting palpebral fissures, thin nasal bridge with pinched tip and hypoplasia of nasal ali, narrow palate, overcrowding of teeth, flat feet, and mild abnormalities of joints of the feet similar to residual effects of prenatal club feet. Creatine kinase was normal.

Their father's uncle was affected by DCM before 18 years old; their paternal grandfather was said to have a “big heart,” and he received PMK over the age of 70 years.

In both of them, genetic analysis identified a paternally inherited heterozygotic variant c.598A>G of *LMNA*, determining the variant p.Met200Val.

### Patient 6

At birth, this patient received a diagnosis of aortic coarctation and was operated on during his first month of life. The patient was regularly seen at our outpatient clinic. Revision of ECG records was normal until age 17 years when he showed polymorphic PVC with mild palpitations. Blood pressure Holter monitoring showed mild hypertension that was treated with atenolol 25 mg, once daily. At 24 years old, an echocardiogram showed an LV at the upper normal limits and a redundant mitral valve apparatus. One year later, he developed a mild dilatation of the ascending aorta and of the sinotubular junction. The ECG Holter monitoring revealed also polymorphic PVC and SVT. At the time of paper revision, the patient was admitted at our institute (aged 28 years) for persistent palpitations and presyncope. There was evidence of ventricular tachycardia. He received ICD implantation according to current guidelines because he had three out of four risk criteria for SCD in laminopathies (non-missense mutation, male gender, ventricular tachycardia) (17). Only once, CK was at the upper normal limits. Facial features included short palpebral fissures, prominent ears, and jaw asymmetry; he had a scoliosis, right-sided hemihypertrophy, and curved vara knees.

Paternal-side family history was significant because his paternal great grandfather died suddenly at 33 years old. His grandfather was affected by DCM and needed PMK implantation. He died at 56 years old. His great uncle had a DCM and a PMK. Great aunt has a history of arrhythmias that the proband parents cannot explain specifically. His father had a DCM and arrhythmias at the fourth decade of life. He was successfully heart transplanted. His uncle was affected by Down syndrome and DCM. Another uncle had DCM and ventricular arrhythmias and had a PMK ICD.

Genetic analysis identified the paternally inherited heterozygotic variant c.214delC of *LMNA*, determining the variant p.Arg72Alafs^*^24. Moreover, the CMP panel analysis identified a paternally inherited heterozygotic variant c.2147C>T of the *ACTN2*, determining the variant p.Thr716Met.

## Discussion

This study is a short case series of patients with laminopathy presenting predominantly in childhood, but more data are needed to delineate this aspect. To the best of our knowledge, this is the first described experience of pediatric cardiogenetic screening with an NGS panel containing *LMNA* in a cohort of children affected by heterogeneous cardiac phenotypes including CMP, CHD, and arrhythmias. Our experience shows the rarity of a clear manifestation of ventricular dysfunction, but it throws the light on previously unexplored aspects of laminopathies, which is the potential aortic involvement in this field.

Our cohort includes six patients (five families) with laminopathies (Table 1). Three of them had a paternally inherited *LMNA* variant; two of them are maternally inherited, and in one case, it was a *de novo* variant.

The variants p.Arg225Ter, p.Arg401Cys, and p.Arg249Gln were previously reported (18–20); p.Met200Val was classified as likely pathogenic, and p.Arg72AlafsTer24 was classified as pathogenic, according to the American College of Medical Genetics guidelines. In patients 1, 2, 4, 5, and 6, the variants showed matched genotype–phenotype correlation in affected family members.

Moreover, our data confirmed previous studies reporting that the arrhythmic events anticipate the myocardial involvement: in patient 1, AF preceded the diagnosis of a mild biventricular dysfunction; in patient 2, malignant arrhythmias preceded the progression to CMP (LVNC with mild LV dysfunction); in patient 3, an EAT was the first manifestation of a DCM, and in patient 6, PVCs and SVT were at least concomitant to the diagnosis of a mildly dilated LV. Patients 4 and 5 had no major arrhythmic events.

Literature review of pediatric laminopathies with cardiac involvement shows six studies with both neuromuscular and cardiac involvements and only one with isolated cardiac abnormalities. When present, the neurologic involvement was the prevalent feature leading to the final diagnosis. Regarding the cardiologic aspect, arrhythmias were the most prevalent pattern of cardiac involvement. Pasqualin et al. (21) described two patients in which one of them died of cardiac arrest due to malignant arrhythmias (6 years old) (p.Asn39Ser), and another one with PVCs (8 years old) (p.Asn39Ser). Komaki et al. (22) described eight pediatric patients with cardiac involvement, three of them at age range of 7–9 years old with heart failure (p.Arg249Gln, p.Leu292Pro, p.Arg377Cys). The other patients had arrhythmic complications without major ventricular dysfunction (pAsn39Asp, p.Arg249Gln, p.Arg28Gln, p.Arg41Ser, p.Arg249Trp) (22). In the report of Petillo et al. (23) only one patient (p.Leu35del) had first-degree AVB and SVT.

Heller et al. (24) reported three cases in which the initial history of bradyarryhthmias/tachyarrhythmias was associated with reduced EF in two cases (13 and 14 years old) and into a restrictive CMP in another case (12 years old) (p.Leu35Pro, p.Arg249Trp, p.Leu380Ser). Parent et al. (25) described two siblings of 9 and 15 years old with DCM-LVNC and LVNC carrying LMNA (p.Arg644Cys) variant without neuromuscular involvement. In the cases reported by Bonne et al. (26), including majorly neuromuscular cohort, five children had arrhythmias, and one of them had associated LV dysfunction (p.Arg249Gln, p.Lys261del, p.Arg386Lys, p.Thr528Lys). In the pediatric population examined by Tan et al. (27), only one patient had ventricular arrhythmias (p.Glu384Gly).

Considering our experience, we highlight the association of laminopathies with CHD and progressive aortic abnormalities in four of our patients. In particular, the first patient had aortic coarctation, bicuspid aortic valve, and a mitral valve cleft; at the age 18 years, he developed AF and mild biventricular dysfunction. The second patient was successfully operated on for a VSD; after 9 years of wellness he survived at a VF, ICD was implanted, and he continued to have malignant arrhythmias: LV function declined to EF 45% at the last observation. The last patient was operated on for aortic coarctation during the first month of life. Many years later, an echocardiogram showed an LV at the upper normal limits, a redundant mitral valve apparatus, a mild dilatation of the ascending aorta and of the sinotubular junction, ectopic ventricular beat, and SVT. Patient 4 had a mildly dilated LV with apical hypertrabeculation but with normal systolic function; he also had a dilated aortic root and an ascending aorta at high limits of the normality.

Identifying the genetic origin of a specific cardiac phenotype is not so straightforward. The interpretation of CMP/rhythm disturbance phenotypes and *LMNA* variants is well-known. However, left-sided CHD phenotype interpretation is more challenging and might not be related merely to a monogenic but rather oligogenic disease form. That is why our cohort was analyzed through NGS custom panel for excluding potential double-hit mutations within another “known” gene causative of CHD. However, we did not identify any of them. It is well-known that all current researches show a limited detection rate in applying NGS panels for CHDs.

Our findings can be explained by the fact that lamin is normally expressed in cardiac tissue, as well as in the vascular walls. Grewal et al. (28) demonstrated a remarkable difference in aortic wall structure and maturation in the presence of bicuspidy, persisting in dilated aortic wall. The major observed changes included a thinner intima, expression at a lower level of a smooth muscle actin, smooth muscle 22α, calponin, and almost absence of expression of smoothelin. They described for the first time a significantly lowered lamin A/C expression in bicuspidy as well (28).

The association of a severe and diffuse aortic hypoplasia and its major branches and Emery–Dreifuss muscular dystrophy (p.Arg249Gln) has been reported only once in literature in a 26-years-old man, screened for heart failure (20). This association has not been reported in previous cohorts. Recent multicentric study reviewed the cardiac and neurologic involvement of laminopathies, concluding that most patients show neurologic symptoms by their fourth decade and develop cardiac involvement at the following decade (29). Our cohort is a bit in contrast to these data. However, the limited number of patients makes it difficult to derive specific conclusions. Pediatric multicentric studies are needed to better explain this rare condition in children.

## Conclusions

Our experience reports the importance of investigating in childhood cohorts laminopathies as a possible cause of progressive LV dysfunction/DCM or bradyarryhthmias/tachyarrhythmias even in the absence of clear neuromuscular involvement and infrequent increase in CK. Paroxysmal AF occurred in 50% of adolescent/young adult patients. Family history integration in NGS era is still a valid tool in guiding clinicians to appropriate and individualized investigations. Furthermore, our analysis highlights a potential causative role of LMNA variant in left-sided CHD and progressive aortopathies (67% of patients): aortic coarctation (two patients), aortic root dilatation (one patient), and VSD (one patient).

Laminopathies have been traditionally described in association with cardiac arrhythmias and DCM and extremely rarely to progressive aortopathies/CHD.

It might be prudent, especially in children with *LMNA* variants, to be investigated for vascular hypoplasia and aortopathies particularly in the context of DCM and arrhythmias. However, it is difficult to derive conclusions from a single study, and further larger and multicentric studies are essential for conclusions.

## Data Availability Statement

The datasets generated for this study can be found in the Bambino Gesù Children's Hospital.

## Ethics Statement

The studies involving human participants were reviewed and approved by Bambino Gesù Children's Hospital. Written informed consent to participate in this study was provided by the participants' legal guardian/next of kin.

## Author Contributions

AB and MC drafted the manuscript and confirmed final version and they are clinicians who cared for the children. MM, MG, and AN were molecular geneticist who undertook the molecular analysis. NC, MS, RA, and FD were clinicians who cared for the children. BD and EB drafted and corrected the manuscript. All authors contributed to the article and approved the submitted version.

## Conflict of Interest

The authors declare that the research was conducted in the absence of any commercial or financial relationships that could be construed as a potential conflict of interest.

## Abbreviations

AF: atrial fibrillation
AVB: atrial-ventricular block
CMP: cardiomyopathy
CK: creatine kinase
CMR: cardiac magnetic resonance
DCM: dilated cardiomyopathy
EAT: ectopic atrial tachycardia
ECG: electrocardiogram
EF: ejection fraction
EMB: endomyocardial biopsy
EVB: ectopic ventricular beats
HF: heart failure
ICD: implanted cardiac defibrillator
*LMNA*: laminin
LV: left ventricle
LGE: late gadolinium enhancement
LVNC: left ventricle non-compaction
NGS: next generation sequencing
NMD: neuromuscular disease
PMK: pacemaker
PVC: premature ventricular complex
RBBB: right bundle-branch block
SCD: sudden cardiac death
SVT: supraventricular tachycardia
VSD: ventricular septal defect
VT: ventricular tachycardia
VF: ventricular fibrillation
Vous: variant of unknown significance.
